# Effect of aging on the association between ankle muscle strength and the control of bipedal stance

**DOI:** 10.1371/journal.pone.0223434

**Published:** 2019-10-03

**Authors:** Zdenek Svoboda, Lucia Bizovska, Zuzana Gonosova, Petr Linduska, Zuzana Kovacikova, Nicolas Vuillerme

**Affiliations:** 1 Department of Natural Sciences in Kinanthropology, Faculty of Physical Culture, Palacky University Olomouc, Olomouc, Czech Republic; 2 AGEIS, Université Grenoble Alpes, Grenoble, France; 3 Institut Universitaire de France, Paris, France; University of Colorado Boulder, UNITED STATES

## Abstract

Previous studies reported a significant association between postural control and lower-limb strength of several muscle groups, however, they were focused especially on knee muscles and ankle plantar and dorsal flexors. The aim of the present study is to examine the correlation between the muscle strength of ankle invertors, evertors, plantar flexors, and dorsal flexors and the control of bipedal stance in young and older adults. Thirty one young (aged 22.8 ± 2.6 years) and thirty one older adults (aged 70.5 ± 7.2 years) voluntarily participated in this study. Ankle muscle strength was evaluated by an isokinetic dynamometer. Normalized peak torque and work were averaged for four repetitions and for both lower limbs. The control of bipedal stance was evaluated by the sample entropy derived from an accelerometer placed on the lumbar spine while the subject stood on a foam pad with eyes open. Results showed significant age-related differences in ankle muscle strength and sample entropy in medial-lateral direction. More interestingly, the correlation between ankle muscle strength and the sample entropy was significantly different between young and older adults. Indeed, no significant correlation was observed in the younger adults. Conversely, in the older adults, the work of the ankle evertors positively correlated with sample entropy in the medial-lateral direction during bipedal stance (*r* = 0.36), whereas the peak torque and work of the dorsal flexors were significantly correlated with sample entropy in the anterior-posterior direction during bipedal stance (*r* = 0.44 for both variables). In the young adults, results suggest that, standing on foam with eyes open is a relatively easy postural task that does not require the full ankle muscle strength capacity. Taken together, the present findings suggest that older adults have a different association between ankle muscle strength and the sample entropy during bipedal stance.

## Introduction

The maintenance of balance is a complex process, and it is influenced by sensorimotor functions and muscle strength, which contribute to joint stabilization. Previous studies reported a significant correlation between postural control and lower-limb strength. Among the most common measures of lower-limb muscle strength is the knee extension strength [1]. Impaired postural control is considered as a risk factor for falls for older adults [2]. Many studies reported a significant correlation between knee extension and postural control in both older men and women [3–5]. In knee osteoarthritis patients, a combination of concentric and eccentric knee flexion and knee extensor strength accounted for approximately 19% of the variability in dynamic balance tests evaluated by the excursion of the center of pressure in the anterior-posterior direction during a forward and subsequent backward lean. [6].

Some attention has been focused also on the ankle joint, especially on the dorsal flexors and partially on the plantar flexors. The strength of these muscles significantly correlates with maximal anterior-posterior displacement of center of pressure [7]. Because the ankle is the bearing joint closest to the body’s support base, it is expected to play a crucial role in maintaining and recovering balance during standing [8–10]. Dorsal flexors control backward slips to prevent the center of mass from moving posteriorly beyond the support base, whereas the plantar flexors act to prevent the center of mass from moving anteriorly beyond the support base [11]. Both dorsal and plantar flexors maintain stability especially in the anterior-posterior direction. Interest has also been focused on ankle muscle groups associated with medial-lateral stability, namely ankle evertors and invertors.

The findings of Alfieri et al. [12] suggest that multisensory exercise programs can improve both postural control and muscle strength of invertors and evertors. Other studies showed that postural control training strengthens the invertors and evertors [13], and that strength training besides improved muscle strength of all ankle muscle groups results in improved postural control [14]. In addition, greater use of ankle evertors owing to their improved strength could be associated with a reduction in mediolateral sway during sufficiently demanding postural tasks [15]. These findings suggest that the muscle strength of not only dorsal and plantar flexors but also invertors and evertors can be associated with level of postural control.

From some studies, it seems that the correlation between ankle muscle strength and the control of bipedal stance is influenced by other factors of the individuals such as age, and from the task and the demands of the environment. In children, no significant correlation was observed between both static and dynamic balance and strength of the plantar flexors [16]. In healthy young adults, results showed no significant correlation between sample entropy and the ankle eversion-to-inversion strength ratio [10]. In older adults, some studies showed significant correlation between strength of dorsal flexors and plantar flexors and selected postural tasks such as tests of limits of stability [7] and one legged stance [17]. Thus, we can expect an aging effect on the correlation between ankle muscle strength and postural control. Similarly, the effect of sensorimotor conditions on the correlation between lower-limb muscle strength and postural control was shown. The main sensorimotor factor contributing to balance under normal conditions (standing on a firm surface with eyes open or closed) is lower limb sensation, whereas during standing on foam surface, but not a firm surface, the sensation of lower limbs is significantly reduced and vision and strength play major roles [5]. In addition, Strang et al. [18] reported that postural control during standing on a foam surface was significantly influenced by balance training, whereas during standing on a firm surface was not. From this point of view, the assessment of postural control during standing on foam seems to be optimal for evaluating the correlation between muscle strength and postural control.

In the past, the control of bipedal stance has been evaluated using especially variables focused on the magnitude of center of pressure movement or acceleration of the body; however, these measures usually focused on the gross amount of movement and ignored the temporal structure of the movement [19]. Non-linear measures such as sample entropy better describe more automatic and a less constrained mode of postural control [20]. In other words, a decrease in sample entropy indicates a more rigid postural behavior, which is considered as dysfunctional postural control [21]. Yamagata et al. [22] reported that older adults use body rigidity to maintain postural stability as a compensative strategy. Furthermore, studies showed that the sample entropy during a postural task varies between different age groups of older subjects [23] or different cognition demands [24]. Finally, several studies showed that in comparison with traditional center of pressure variables, entropy measures can better predict future falls in older adults [25], are able to distinguish between older subjects with and without history of lateral ankle sprain [26], and are able to assess the effects of Tai Chi training on standing postural control [27].

To the best of our knowledge, no study has assessed the correlation between control of bipedal stance and muscle strength of plantar flexors, dorsal flexors, invertors, and evertors. Furthermore, any effect of aging on this correlation is still not clear. Along these lines, the aim of this study was to assess the correlation between the muscle strength of ankle invertors, evertors, plantar flexors, and dorsal flexors and the control of bipedal stance in young and older adults. We hypothesized that there is higher correlation between muscle strength and sample entropy in older compared to young adults.

## Materials and methods

### Research sample

We recruited the younger subjects among university students, the older subjects through the University of the Third Age at Palacký University Olomouc (Czech Republic), through a local seniors’ magazine, or through personalized invitation letters. Inclusion criteria for participation in the study were age (young group: 18 to 30 years, older group: 60 to 90 years), the ability to walk without an assistive device, and the ability to stand without any support during common everyday activities. Exclusion criteria were neurological or vestibular diseases and surgery in lower limbs or spine during the last two years. The research sample consisted of 31 young adults (18 females and 13 males, age: 22.8 ± 2.6 years, height: 170.2 ± 8.3 cm, body mass: 67.9 ± 10.7 kg) and 31 older adults (24 females and 7 males, age: 70.5 ± 7.2 years, height: 166.1 ± 8.4 cm, body mass: 75.7 ± 14.2 kg). All participants provided written statements of informed consent, and the testing protocol was approved by the Ethics Committee of the Faculty of Physical Culture, Palacký University Olomouc.

### Procedures

#### Postural control

Participants stood barefoot on a foam pad (Airex Balance Pad, Airex AG, Sins, Switzerland) in a comfortable bipedal stance with their arms along their sides. We instructed the participants to stand as still as possible and to fix their gaze on a circular mark (diameter 5 cm) located two meters in front of them on the wall at eye level. A 3D accelerometer (Trigno wireless system, Delsys Inc., Natick, MA, USA; sampling rate 148 Hz) we securely attached to the participant’s lower back at the level of the fifth lumbar vertebra. The acceleration signals in the medial-lateral and anterior-posterior directions we recorded in each trial. Each trial lasted 30 s with recording starting after 2 to 3 s of stabilization. Three repetitions of the trials were performed with sufficient time for rest between them. We filtered the acceleration signals using a fourth-order low-pass bidirectional Butterworth filter with a cutoff frequency of 10 Hz. As an indicator of postural control, we computed sample entropy from acceleration signals for each time series, with the number of consecutive points *m* set to 2 and the similarity criterion *r* set to 0.15 [28–31] independently for the medial-lateral and anterior-posterior directions. We performed data analysis using MatLab software (R2018a, MathWorks, Inc., Natick, MA, USA).

#### Muscle strength assessment

The isokinetic strength of the lower limbs we measured using an IsoMed 2000 isokinetic dynamometer (D. & R. Ferstl GmbH, Hemau, Germany). A nonspecific warm-up was performed before the isokinetic testing under the supervision of the researcher. The warm-up consisted of 5 min of cycling on a stationary Kettler ergometer (Heinz Kettler GmbH & Co. KG, Ense-Parsit, Germany) at a submaximal intensity of 1.0 W/kg of body mass and a pedal rate of 60–70 rpm, followed by 10 min of dynamic stretching exercises of lower-limb muscles. Immediately after the nonspecific warmup, participants completed isokinetic testing of the muscle strength of the ankle invertors and evertors and the ankle plantar and dorsal flexors. Positioning and securing of the participants on the adjustable isokinetic dynamometer we conducted in agreement with the manufacturer’s recommendations. Before the test, we calibrated the device according to the manufacturer’s instructions. Participants underwent an isokinetic familiarization session one week prior to the study.

Positioning for the ankle inversion and eversion was performed as follows. The participants were seated on the isokinetic dynamometer seat with the hip joint at approximately 80° and the knee at 110° (180° = full knee and hip extension), such that the shin was positioned horizontally to the ground. We placed the foot on the foot adapter with an ankle angle of 10° of plantar flexion (0° = neutral position of the talocrural joint) and secured with two Velcro straps. We used a handheld goniometer to set the angles. The hip and knee joint angles we adjusted by changing the distance between the chair and the foot adapter and the height of the foot adapter. The waist we secured by means of straps. There was a support adapter under the knee of the tested limb and the thigh we secured with straps. We secured also the shoulders in the ventral–dorsal and cranial–caudal directions by shoulder straps and pads. We flexed the contralateral limb to approximately 90° in the knee joint and we placed the foot on the table. We set the range of motion from 25° of the ankle eversion to 20° of the ankle inversion (0° = neutral position of subtalar joint).

Positioning for the ankle plantar and dorsal flexion we performed as follows. We laid the participants supine on the isokinetic dynamometer table with hip and knee in full extension. We placed the foot on the foot adapter connected to the head of the dynamometer and secured with two Velcro straps. The axis of rotation we aligned with the axis of rotation of the lateral malleolus. The thigh of the tested limb and the waist we secured by means of straps, and an overball we placed underneath the knee of the tested limb to prevent overextension of the knee and for the subject’s comfort. The shoulders we secured in the ventral–dorsal and cranial–caudal direction by shoulder straps and pads. The contralateral limb we flexed to approximately 90° in the knee joint and the foot we placed on the table. After the securing, we applied a gravitational correction. The range of motion we set from 10° of the ankle dorsal flexion to 35° of the ankle plantar flexion (0° = neutral position of talocrural joint).

Isokinetic testing we conducted at a velocity of 30°/s, which is in the agreement with the range of velocities typically studied for the ankle joint in elderly subjects [17]. Prior to each test, participants performed three to five submaximal practice trials in tested movements as a specific warm-up to become acquainted with the requirements of the test. Afterward, a test of four maximal efforts was conducted. There were rest periods of 2 min between tests of the two lower extremities and 5 min between tested movements. We instructed each subject to exert maximal voluntary effort as hard and as fast as possible throughout the entire range of motion. We notified the participants by a verbal countdown, and gave them strong verbal encouragement and visual real-time feedback of torque development to ensure maximal effort. After testing the first lower limb, we tested the second lower limb according to the same procedure, during which individual settings were automatically activated, rechecked, and adjusted if necessary.

For data recording and reduction, we used the manufacturer’s computer software IsoMed Analyze (version 1.0.5, D. & R. Ferstl GmbH, Hemau, Germany). We extracted values of peak torque [N.m.kg^-1^] and work [J.kg^-1^] normalized by body mass for each movement. For each analysis, we considered the dominant and non-dominant limbs separately, and then we computed the means of the variables obtained for each limb.

### Statistical processing

All statistical procedures were performed using Statistica software (version 12, StatSoft, Inc., Tulsa, OK, USA). Normal data distributions were verified by the Kolmogorov-Smirnov test. For comparison of groups with different ages, the independent *t*-test was used. The significance level was stated as α = 0.05. We applied a Holm-Bonferroni correction to counteract the problem of multiple comparisons [32]. A correction factor was used for groups of sample entropy and muscle strength parameters as follows: sample entropy (smallest p-value = 0.05/2 = 0.025), muscle strength (smallest p-value = 0.05/8 = 0.00625). The effect size was evaluated using Cohen’s *d*. Values of 0.2, 0.5, and 0.8 for Cohen’s *d* were considered to show a small, medium, and large effect, respectively. The correlation between sample entropy and muscle strength variables was obtained using a Pearson correlation.

## Results

Compared to the older adults, the young adults exhibited significantly higher sample entropy in both medial-lateral and anterior-posterior directions, and significantly higher muscle strength in all observed muscles (Table 1).

**Table 1:** Sample entropy and normalized muscle strength in various ankle muscle groups (mean±SD).

Variable		Young	Older	*p*	Cohen’s *d*
Sample Entropy	Medial-lateral	0.54 ± 0.06	0.48 ± 0.08	0.004	0.77
	Anterior-posterior	0.38 ± 0.09	0.31 ± 0.09	0.006	0.73
Invertors	Peak torque	0.34 ± 0.08	0.21 ± 0.05	< 0.001	1.94
	Work	0.19 ± 0.05	0.12 ± 0.03	< 0.001	1.85
Evertors	Peak torque	0.32 ± 0.08	0.19 ± 0.05	< 0.001	2.00
	Work	0.18 ± 0.05	0.10 ± 0.03	< 0.001	2.01
Plantar flexors	Peak torque	1.61 ± 0.34	0.93 ± 0.27	< 0.001	2.20
	Work	0.83 ± 0.17	0.46 ± 0.14	< 0.001	2.38
Dorsal flexors	Peak torque	0.42 ± 0.08	0.33 ± 0.08	< 0.001	1.19
	Work	0.24 ± 0.05	0.18 ± 0.05	< 0.001	1.12

Peak torque [N.m.kg^-1^], Work [J.kg^-1^]

Results further showed no significant correlation between sample entropy and ankle muscle strength in the young adults. Conversely, in older adults, the muscle strength of ankle evertors (work) was significantly positively correlated with sample entropy in the medial-lateral direction (Table 2). In addition the muscle strength of dorsal flexors (both peak torque and work) was significantly positively correlated with sample entropy in the anterior-posterior direction (Table 3).

**Table 2:** Correlations between ankle muscle strength (inversion and eversion) and sample entropy in medial-lateral direction.

Variable		Young		Older	
		*R*	*p*	*r*	*p*
Invertors	Peak torque	0.00	0.986	0.16	0.377
	Work	0.08	0.657	0.29	0.108
Evertors	Peak torque	0.07	0.689	0.12	0.510
	Work	0.16	0.402	0.36	0.046

Peak torque [N.m.kg^-1^], Work [J.kg^-1^]

**Table 3:** Correlations between ankle muscle strength (plantar and dorsal flexion) and sample entropy in anterior-posterior direction.

Variable		Young		Older	
		*r*	*p*	*R*	*P*
Plantar flexors	Peak torque	-0.12	0.523	0.19	0.309
	Work	-0.19	0.294	0.20	0.288
Dorsal flexors	Peak torque	0.21	0.261	0.44	0.013
	Work	0.21	0.261	0.44	0.013

Peak torque [N.m.kg^-1^], Work [J.kg^-1^]

## Discussion

The purpose of this study was to assess the correlation between muscle strength of the ankle muscles and the sample entropy during bipedal stance in young and older adults. For postural control assessment, we computed sample entropy derived from accelerometers placed on the lumbar spine, which is considered an outcome that better characterizes more automatic and less constrained modes of postural control [20–24].

Comparison of young and older adults demonstrated that there is a significant effect of age on ankle muscle strength. Our results support the existing literature [33–34]. Significant differences in muscle strength had been found between the young (mean age 23 years) and older (mean age 77 years) participants in all ankle muscle groups, with older participants being weaker than the young participants by approximately 30% [34]. In our study, the decline of peak torque in older (mean age 71 years) compared to young adults (mean age 23 years) was 38% for invertors, 40% for evertors, 42% for plantar flexors, and 23% for dorsal flexors. The reason for these changes could be the normal aging process associated with worse function of neuromuscular system [35].

Our study further demonstrated a decrease in sample entropy during control of bipedal stance in older subjects compared to young ones, in both medial-lateral and anterior-posterior directions. Similar findings for the anterior-posterior direction have been reported recently by Potvin-Desrochers et al. [36]. For the medial-lateral direction, these authors did not find any significant difference between young and older adults. In another study focused on adolescents, the age was significantly correlated with higher sample entropy only in the medial-lateral direction [37]. Other studies indicated that restricted vision and a compliant surface represent constraints to postural control that cause a decrease in sample entropy [17]. On the other hand, a simple cognitive task can increase sample entropy in both young and older adults, because performing a concurrent cognitive task promotes the adoption of an automatic postural control [36]. Our results suggest that, when older individuals stand on a foam surface, these participants maximize the use of multiple sources of information in comparison to young adults and have problems selecting and integrating information properly [38]. This results in an increased need for attention and thus a decreased automaticity.

Observing which factors can influence postural control is very important, especially due its relation to fall risk. One of the factors is considered to be lower-limb muscle strength. In the past, the main focus of scientific studies has been the strength of knee extensors and ankle dorsal flexors [1]. Although other ankle muscles are also often considered as important for postural control during standing, the number of studies concerning these muscles is very limited. Especially in ankle evertors and invertors, lack of studies could be associated with difficulties during testing of these muscles. However, in past years, good reliability of ankle muscle strength measurements including ankle invertors and evertors using isokinetic measurement devices has been reported [39].

Our results showed no significant correlation between ankle muscle strength and sample entropy in the young adults. Similarly in the scientific literature, no significant correlation between postural control (center of pressure displacement in medial lateral and anterior posterior directions) and strength of plantar flexors was found in children [16], nor between postural control (mean center of pressure velocity, area of confidence ellipse) and the ankle eversion-to-inversion strength ratio in young adults [10]. On the other hand, significant correlations between postural control evaluated by sample entropy in the anterior-posterior direction and peak torque and work of dorsal flexors were found in our study in the older subjects. Wiksten et al. [17] found a significant correlation between duration of stance ask and average torque of dorsal flexors, but only for a one-leg stance on the non-dominant limb with eyes closed, not for a bipedal stance. Similarly, Melzer et al. [7] found no significant correlation between muscle strength of dorsal flexors and postural control during a bipedal stance (center of pressure maximal displacement, mean velocity, sway area), but found a significant correlation in a test of limits of stability. Taken together, these results and ours suggest that the presence or absence of a significant correlation between muscle strength and postural control could be related to postural demands associated with the postural task.

In addition, our study also indicates a significant correlation between muscle work of ankle evertors and sample entropy in the medial-lateral direction. This result suggests that these muscles have important effect on postural control in medial-lateral direction. In the scientific literature there is lack of evidence focused of relationship between ankle evertors muscle strength and postural control in older adults. Some studies focused on subjects with ankle instability showed that these subjects can be characterized by both weakness of evertor muscles and decreased postural control in medial lateral direction [40]. So our data suggest that also in older adults weaker ankle evertors can result in increased postural sway during standing on a foam pad.

It is interesting that although maximal muscle strength is not necessary for maintaining stability during bipedal stance, our results showed association between maximal strength of ankle muscles and postural sway during postural tasks. It seems that increased maximal strength is associated with greater capacity of muscles and possibility to increase muscle activity of the ankle muscles even during relatively simple postural tasks resulting in reduction of postural sway [15]. Other studies showed that strength training in older adults results in improvement of both neuromuscular performance and functional capacity of the subjects [41]. On the other hand these correlation wouldn’t be overestimated. Coefficients of determination (r^2^) derived from correlation coefficients show that muscle strength of ankle dorsal flexors and ankle evertors can explain only 19% or 13% of proportion of the variance of sample entropy during postural task.

The previously mentioned results indicate that the correlation between ankle muscle strength and postural control is influenced by age. This result supports those by Wiksten et al. [17]. Indeed, these authors found significant correlations between lower-limb muscle strength and postural control only in older individuals. In general, we can say that correlation coefficients for young adults are smaller compared to those for children and older groups [42]. It seems that the muscle strength capacity in young adults is relatively greater than the muscle strength requirement for a bilateral-stance postural task.

As limitation of this study we consider higher participation of women. However, the present study is focused especially on exploring correlations, and these aspects probably do not influence the findings considerably.

## Conclusion

Taken together, the present findings suggest that aging has an effect on the correlation between ankle muscle strength and the control of bipedal stance. By showing significant correlations between ankle muscle strength and the postural control during bipedal stance in older adults, these results could have relevant implications for future implementation of exercise programs for improving postural control in older people.

## Supporting information

S1 Dataset This is the dataset. (XLSX)
